# C3 glomerulopathy: a kidney disease mediated by alternative pathway deregulation

**DOI:** 10.3389/fneph.2024.1460146

**Published:** 2024-10-29

**Authors:** Karin Heidenreich, Deepti Goel, P. S. Priyamvada, Sagar Kulkarni, Vipul Chakurkar, Dinesh Khullar, Ravi Singh, Charan Bale, Peter F. Zipfel

**Affiliations:** ^1^ eleva GmbH, Freiburg, Germany; ^2^ Clinical Programs, Mumbai, India; ^3^ Department of Nephrology, Jawaharlal Institute of Postgraduate Medical Education and Research, Puducherry, India; ^4^ Department of Nephrology, King Edward Memorial Hospital, Pune, Maharashtra, India; ^5^ Department of Nephrology and Renal Transplantation, Max Super Speciality Hospital Saket, New Delhi, India; ^6^ Department of Nephrology and Renal Transplant, Jaypee Hospital, Noida, Uttar Pradesh, India; ^7^ Department of Nephrology, Dr. D.Y. Patil Medical College & Research Centre, Pune, Maharashtra, India; ^8^ Department of Infection Biology, Leibniz Institute for Natural Product Research and Infection Biology, Hans Knöll Institute, Jena, Germany

**Keywords:** C3 glomerulopathy, complement, therapy, complement inhibitors, alternative pathway (AP)

## Abstract

C3 glomerulopathy (C3G) is an ultra-rare complement-mediated kidney disease caused by to the deregulation of the alternative pathway (AP) of proximal complement. Consequently, all effector loops of the complement are active and can lead to pathologies, such as C3a- and C5a-mediated inflammation, C3b opsonization, surface C3b-mediated AP C3 convertase assembly, C3 cleavage product deposition in the glomerulus, and lytic C5b-9/MAC cell damage. The most common pathologic mechanisms are defective chronic alternative pathway deregulation, mostly occurring in the plasma, often causing C3 consumption, and chronic complement-mediated glomerular damage. C3G develops over several years, and loss of renal function occurs in more than 50% of patients. C3G is triggered by both genetic and autoimmune alterations. Genetic causes include mutations in individual complement genes and chromosomal variations in the form of deletions and duplications affecting genes encoding complement modulators. Many genetic aberrations result in increased AP C3 convertase activity, either due to decreased activity of regulators, increased activity of modulators, or gain-of-function mutations in genes encoding components of the convertase. Autoimmune forms of C3G do also exist. Autoantibodies target individual complement components and regulators or bind to neoepitopes exposed in the central alternative pathway C3 convertase, thereby increasing enzyme activity. Overactive AP C3 convertase is common in C3G patients. Given that C3G is a complement disease mediated by defective alternative pathway action, complement blockade is an emerging concept for therapy. Here, we summarize both the causes of C3G and the rationale for complement inhibition and list the inhibitors that are being used in the most advanced clinical trials for C3G. With several inhibitors in phase II and III trials, it is expected that effectice treatment for C3G will become availabe in the near future.

## 1 Introduction

C3 glomerulopathy (C3G) is a complement-mediated kidney disease caused by dysregulation of the alternative pathway in plasma leading to complement activation with effector actions manifested in the glomeruli (1, 2). C3G is a chronic, slowly progressive disease. C3G presents in two subforms, namely, C3 glomerulonephritis and dense deposition disease, and shows the absence or low levels of deposited immunoglobulins (3–5).

To understand the pathology of the disease and ultimately select the right complement inhibitor for therapy, a detailed understanding of how and where individual genetic alterations and autoimmune factors cause complement dysregulation is relevant. It is also important to know which steps of the cascade are affected and which effector loops are activated. In addition, it is essential to understand how defective complement leads to morphological changes, mesangial proliferation, inflammation, and cell lysis, leading to renal damage. This information will help to define the level at which the complement cascade can best be targeted by inhibitors (6–9).

There is currently no approved disease-specific therapy for C3G. Standard of care includes supportive care, renoprotective therapy with angiotensin-converting enzyme (ACE) inhibitors, or angiotensin receptor blockade for blood pressure control. In addition, immunosuppression with mycophenolate mofetil (MMF) and glucocorticoids is used for moderate and severe disease.

Because C3G is an AP complement disease caused by defective action of the alternative pathway, complement blockade is an emerging concept for therapy. Defective complement can be treated, as has been shown for the renal disease atypical hemolytic uremic syndrome (aHUS), as well as for hematologic, neuronal, and retinal disorders. Several complement inhibitors are currently approved for therapy.

In this review, we focus on five central topics relevant to C3G: i) provide an overview on the pathophysiology of C3G; ii) report which mutations in complement genes and autoimmune causes are described in C3G; iii) show how different genetic alterations and also autoantibodies result in almost identical features: AP overactivation, consumption of C3 in plasma causing the same glomerular alterations, C3b deposition along and thickening of the glomerular basement membrane, and loss of kidney function; iv) provide a brief overview on disease C3G prevalence in India; and v) touch on the promising development of new complements drugs which are currently being evaluated for these important rare kidney diseases.

## 2 Complement

**Figure 1:**
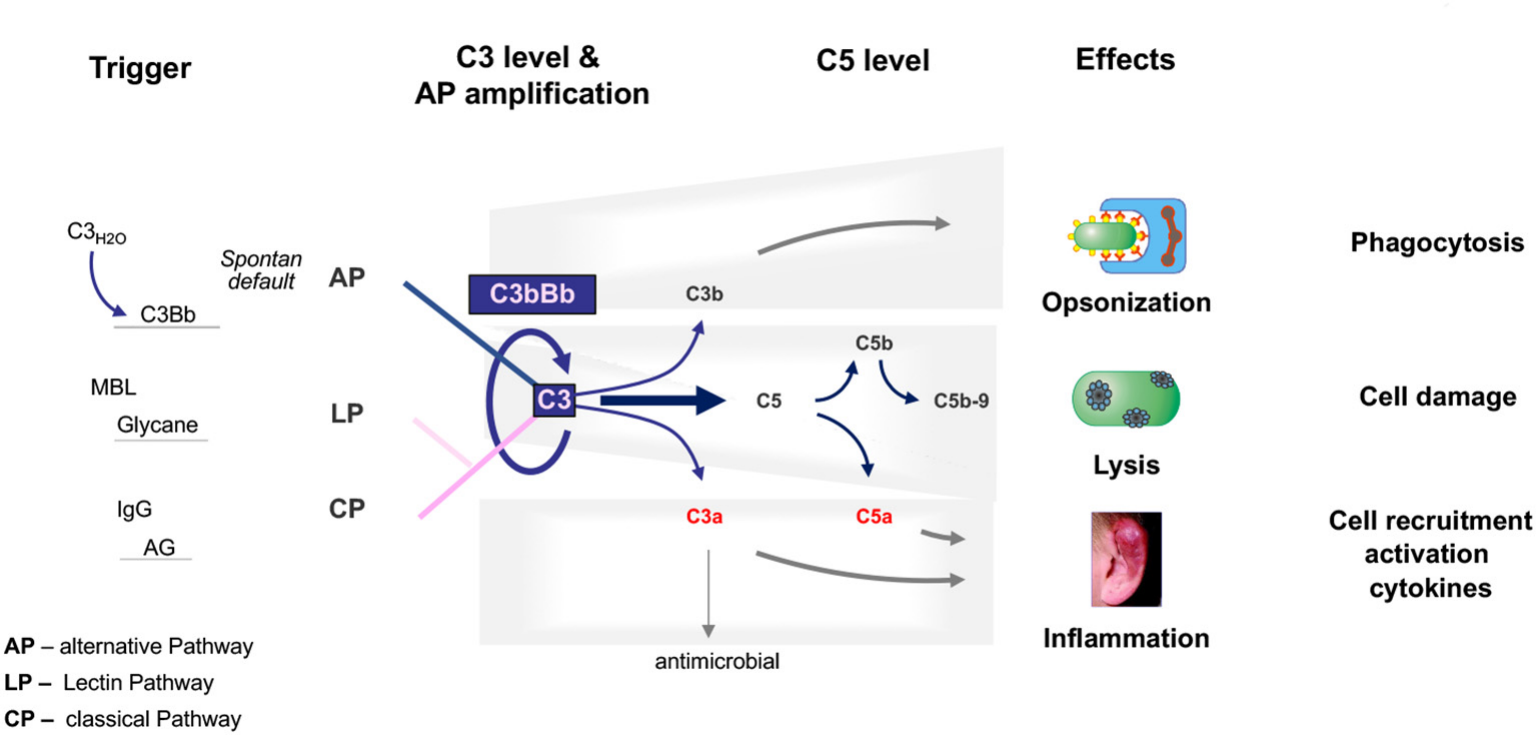
The physiological complement. The complement is activated through three pathways: the alternative pathway, the lectin pathway, and the classical pathway. The C3 convertase of the alternative pathway, which is composed of C3b and Bb, is shown and is at the center of the pathophysiology of C3G. C3 convertases cleave C3 into the anaphylatoxins C3a and C3b. C3a also has antimicrobial activity. C3b decorates, opsonizes target surfaces, and allows phagocytosis of the decorated particle. Complement activation proceeds to the level of C5 convertases, which cleave C5 to the potent anaphylatoxin and inflammatory mediator C5a and to C5b, which can assemble C6, C7, C8, and C9 to form the lytic pore, C5b-9. The triggers for each activation pathway are shown on the left side. The major effector functions of the complement are shown on the right.

The complement system is an essential part of the innate immune system that is critical for defense against infectious microbes and for maintaining homeostasis. The complement system consists of more than 50 plasma proteins and activation products. Most complement proteins are produced and secreted primarily in the liver, and most are expressed on the surfaces of various cell types and many activation products (10, 11). The system is activated by three pathways: the alternative pathway (AP), the classical pathway (CP), and the lectin pathway (LP). The AP, unlike the triggered CP and LP, is permanently active by default (12). The CP is activated by antibody::antigen complexes, and the LP is triggered by surface-expressed carbohydrates (13). The three activation pathways generate C3 convertases that cleave circulating C3 into C3a and C3b. C3a mediates anaphylactic and inflammatory responses, and C3b decorates target surfaces of foreign microbes or modified self-cells, resulting in enhanced non-inflammatory clearance (14–16). Surface-deposited C3b also provides a platform for Factor B assembly, which upon activation to Bb, forms the AP C3 convertase. This enzyme cleaves additional C3 and initiates the AP amplification loop. The cascade can continue to generate C5 convertases that cleave C5 to produce the potent anaphylatoxins C5a and C5b. C5b assembles with C6, C7, C8, and C9 to form C5b-9, also known as the MAC or TCC complex, which forms a lytic pore (17, 18) (**Figure 1**).

Physiological permanent AP tick-over activation occurs at low levels in the fluid phase (13). However, all subsequent steps, fluid-phase amplification, the transition from circulation to surfaces, AP C3 convertase activity, and initiation of the amplification loop are tightly regulated by circulating plasma regulators and membrane-bound inhibitors. This allows the immune system to selectively attack and damage microbial, foreign, and modified self-surfaces while protecting and preserving intact self-tissue.

In C3G, AP dysregulation occurs in the circulation in the plasma and kidney and on surfaces such as the glomerular basement membrane, causing glomerular damage that evolves into C3G pathology.

## 3 C3 glomerulopathy

C3G is a clinical entity resulting from deregulation and consequently overactivation of the AP of complement. Overactivation of the AP pathway occurs mainly systemically in the plasma but also locally in the glomerulus. Within the glomerular microenvironment, a deregulated complement leads to the deposition of C3b on target surfaces. Deregulated and overactive AP C3 convertases allow the cascade to progress and activate all effector levels (19, 20).

In 2014, the C3G classification was introduced to reflect strong glomerular C3b staining in the absence of immunoglobulin reactivity. Previously, the same disease or glomerular patterns were referred to as membranoproliferative glomerulonephritis (MPGN) to reflect the proliferative nature of the mesangial cells and the inflammatory nature. The term type II was used to describe the proliferation of mainly endothelial cells. The electron-dense deposits that form along the glomerular basement membrane (GBM) are thought to be the result of a repair response induced by chronic complement attack (20, 21).

C3G presents challenges in both diagnosis and treatment.

Plasma diagnosis: Many patients with C3G show AP complement activation in the fluid phase, diagnostically evidenced by APH50 activity and low plasma C3 levels. Plasma levels of complement activation fragments Ba, Bb, C3a, and sC5b-9/MAC are also often elevated. Plasma C4 levels, reflecting CP and LP action, are often in the normal range (22).

Age of onset: C3G can affect people of any age although it is most common in children and young adults. Over time, without appropriate treatment and management, patients may progress to kidney damage and even end-stage renal disease (ESRD), requiring dialysis or kidney transplantation (23).

### 3.1 Pathophysiology

In C3G, chronic AP activation occurs in the fluid phase and on surfaces, leading to C3b deposition along the glomerular basement membrane, in the mesangium, and within the subendothelial space (24–27).

C3b deposition along the GBM and subendothelial and mesangial cells results in a membranoproliferative pattern, and continuous deposition of complement products at the GBM can lead to dense deposits. Occasionally, deposits also form on the outer surface of the GBM, resulting in subepithelial deposits. Chronic local complement action leads to the deposition of C3b and the release of anaphylatoxins, which induce cell activation, proliferation, and macrophage infiltration, explaining the mesangial hypercellularity and endocapillary formation. Continuous damage to the GBM induces repair reactions that can lead to the thickening of the GBM, explaining the increase in the mesangial matrix and the formation of double contours.

During such a chronic complement attack, all effector pathways are activated, including anaphylatoxin-mediated inflammation, C3b opsonization, formation of more AP C3 convertase, and C5b-9/MAC-mediated cell damage.

Histologic findings include deposition of C3b and C5b-9/MAC, as well as properdin and staining for the complement modulators FHR1 and FHR5.

C3G has both genetic and autoimmune causes. Mutations in genes encoding complement regulators, modulators, and C3 convertase components are responsible for pathology. In addition, antibodies are directed against various complement proteins, regulators, AP C3 convertase, or C5 convertase contribute to disease. Thus, proximal AP dysregulation induces all effector routes of complement, which results in inflammation and local damage.

Clinical presentation: C3G can present in various ways with nephrotic syndrome being a common presentation. Often, the patients in their second or third decade present with edema, cloudy urine, and fatigue. Proteinuria serves as the primary diagnostic marker, followed by the detection of microscopic hematuria. Hypertension is seen in nearly half of the patients, more so in those presenting in their fifth decade of life. The concomitant or preceding history of infection is sometimes seen, which makes differential diagnosis difficult. It is observed by the authors that many patients in India present late with the disease. It is not uncommon to encounter patients with end-stage kidney disease with small kidneys with persistent low serum C3 levels.

Symptoms: Patients may present with a variety of symptoms including proteinuria, hematuria, and decreased renal function. Also, the estimated glomerular filtration rate is often decreased, and creatinine levels in the blood or urine are elevated.

Diagnosis: Urine analysis is the first step in the evaluation of the disease. In C3G, it shows typically proteinuria and hematuria. The extent of proteinuria must be documented with either a 24-h collection or urine protein:creatinine ratio. Kidney function should be measured at the baseline and monitored during follow up. Often, patients with nephrotic syndrome due to C3G have elevated serum creatinine value at presentation. Serological tests should include serum levels of C3 and C4. In C3G often the level of C3 are persistently low with normal C4 levels. Evidence of infection should be ruled out at the initial presentation. The finding of low C3 levels at 6 weeks of illness often differentiates C3G from post-streptococcal GN in children.

The hallmark and current diagnostic criterion of C3G is immunofluorescence finding of strong C3b deposits along the glomerular, basement membranes, as well as the mesangium, that are two orders of magnitude higher that immunoglobulint. If immunoglobulin deposits are observed, they are polyclonal in most cases, but monoclonality with light-chain restriction is seen when C3G is related to monoclonal disorders. Renal biopsy is essential for diagnosis. The histopathologic features of C3 glomerulopathy are characterized by deposition of C3, the absence or minimal immunoglobulin deposition within the glomeruli, evidence of glomerular inflammation, and mesangial cell proliferation.

C3G is subdivided into C3 glomerulonephritis (C3GN) and dense deposition disease (DDD). Electron microscopy is essential to identify dense deposits and to differentiate C3G with a DDD pattern from C3GN and other forms of glomerulonephritis and to guide the diagnosis. In C3GN, deposits appear to be more prominent in the mesangium and along the subendothelial sites. DDD is characterized by electron-dense deposits along the glomerular basement membrane. These newly formed structures can be viewed as the result of a damage repair continuum (2, 23).

### 3.2 Genetic causes of C3G

The genetic causes of C3G include mutations in the genes encoding the complement proteins Factor H and C3; the five FHR proteins FHR1, FHR2, FHR3, FHR4, and FHR5; C5; Factor B; Factor I; MCP/CD46; clusterin; properdin; and plasminogen (28–30). Chromosomal rearrangements in the FHR gene cluster have also been reported. The mutant or altered proteins cause deregulation of the AP C3 convertase by loss of function, the absence of regulators, or gain of function as described for Factor H, FHL1, C3, Factor B, and FHR::FHR hybrid proteins (31, 32). FHL1, the Factor H-like protein, is encoded by an alternatively spliced transcript of the Factor H gene. FHL1 shares with Factor H the N-terminal seven SCRs (short consensus repeat domains) (33). Each of the five FHR (Factor H-related) proteins is encoded by a unique gene. FHR1, FHR2, FHR3, FHR4, and FHR5 are complement modulators and are associated with several human diseases, and the function of the individual FHR proteins is currently investigated (34). Several FHR::FHR hybrid proteins enhance the activity of the AP C3 convertase resulting in overactive AP, during C3 tick-over activation, for surface-bound AP C3 convertases and for the amplification loop (35). In 20% of cases, C3G is the result of genetic mutation (along with a “second hit”). So, genetic testing for the mutations in genes encoding Factor H, Factor I, C3, FHR 1, FHR2, FHR3, FHR4, and FHR5 is an important part of the workup (33, 36–38). Whole exome sequencing is the preferred method but is likely to miss copy number variations in the complement and related genes (31, 32). The latter is generally picked up by multiplex ligation dependent probe amplification (MLPA) with the FHR gene panel. The presence of C3G in a patient more than 50 years old should also prompt investigations for monoclonal gammopathy.

**Figure 2:**
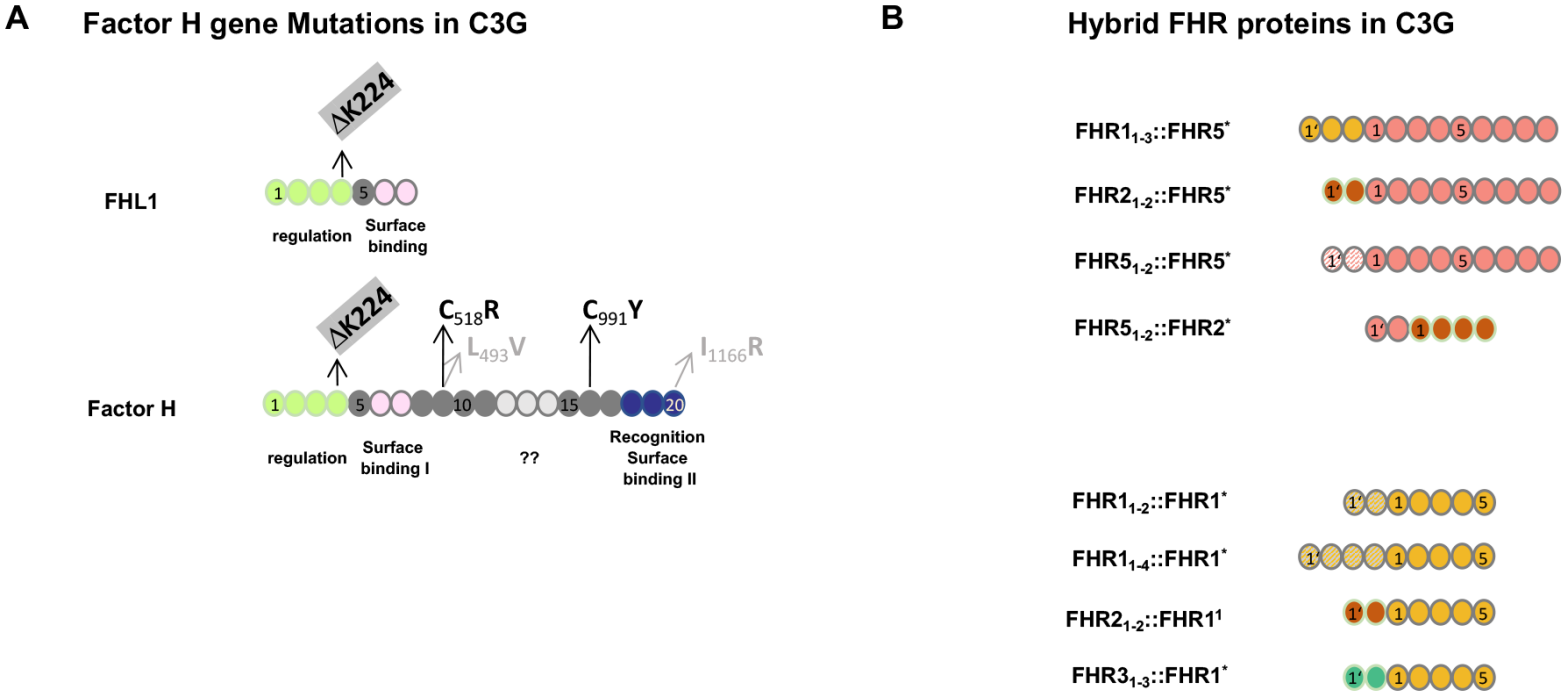
Genetic mutations in Factor H and FHR hybrid proteins associated with C3G. **(A)** Mutation in the *Factor H* gene in C3G patients and in Factor H-deficient pigs. The structure of Factor H and the alternatively spliced product FHL1 is shown. The functional protein regions are shown below the two proteins. ?? indicates that the function of this region is not understood at the moment. Patients #2 and #3 (see Section 3.4.2 below) have a homozygous deletion of residue K224 in SCR4. This K224 deletion results in defective regulation of both Factor H and FHL1 proteins. The two compound heterozygous Factor H mutations in SCR9 and SCR16 identified in the 13-month-old Sioux boy are shown in black. The compound heterozygous exchanges in SCR9 and SCR20 in the Factor H-deficient pigs are shown in gray. **(B)** FHR hybrid proteins identified in familial C3G. The *FHR* gene cluster represents a region of chromosomal instability and is frequently affected by non-homologous recombination. The lower panel shows examples of hybrid proteins, all of which contain an intact FHR1 backbone protein, shown in yellow. In the different scenarios, either N-terminal domains are duplicated or N-terminal SCRs from FHR2 (red) or FHR3 (green color) have been added. The upper panel shows three familial cases in which two or three SCRs were added to the N-terminus of FHR5 (orange). The added domains were the dimerization region of FHR2 (red), FHR5 itself (dashed line), or FHR1 (yellow). The scenario at the bottom is a reverse genetic situation where the N-terminal FHR5 dimerization domain (blue) is attached to the FHR2 protein. Index patients #3 and #4 are shown in the bottom panel in row 1 and row 4, respectively. All scenarios involving FHR gene variations occur in the context of an intact Factor H gene and in the presence of Factor H and FHL1 in the plasma.

Factor H gene mutations have been described in C3G patients and animal models. A 13-month-old child diagnosed with membranoproliferative glomerulonephritis was found to be Factor H-deficient. This boy had compound heterozygous mutations, i.e., in allele 1: cDNA.T1679C, p.C518R in SCR9 and in allele 2: cDNA. G2949A, p.C991Y in SCR16 (39, 40). Both exchanges occur in central framework Cys residues and result in defective protein secretion and the absence of Factor H in the plasma. In C3G patients, additional genetic alterations including mutations in Factor B, FHR1, FHR2, FHR3, FHR4, FHR5, C3, clusterin, properdin, and plasminogen have been described (**Figure 2**) (28–30, 35).

The porcine model, which was actually the first animal model for MPGN II/DDD or C3G, showed the absence of Factor H due to mutations of L493V in SCR9 and I1166R in SCR20. Thus, the exchange of two non-framework residues results in a block of Factor H secretion (41, 42). Factor H knockout mice, which lack Factor H, are well characterized and represent a valuable animal model to study C3G (43, 44).

### 3.3 Autoimmune causes of C3G

**Figure 3:**
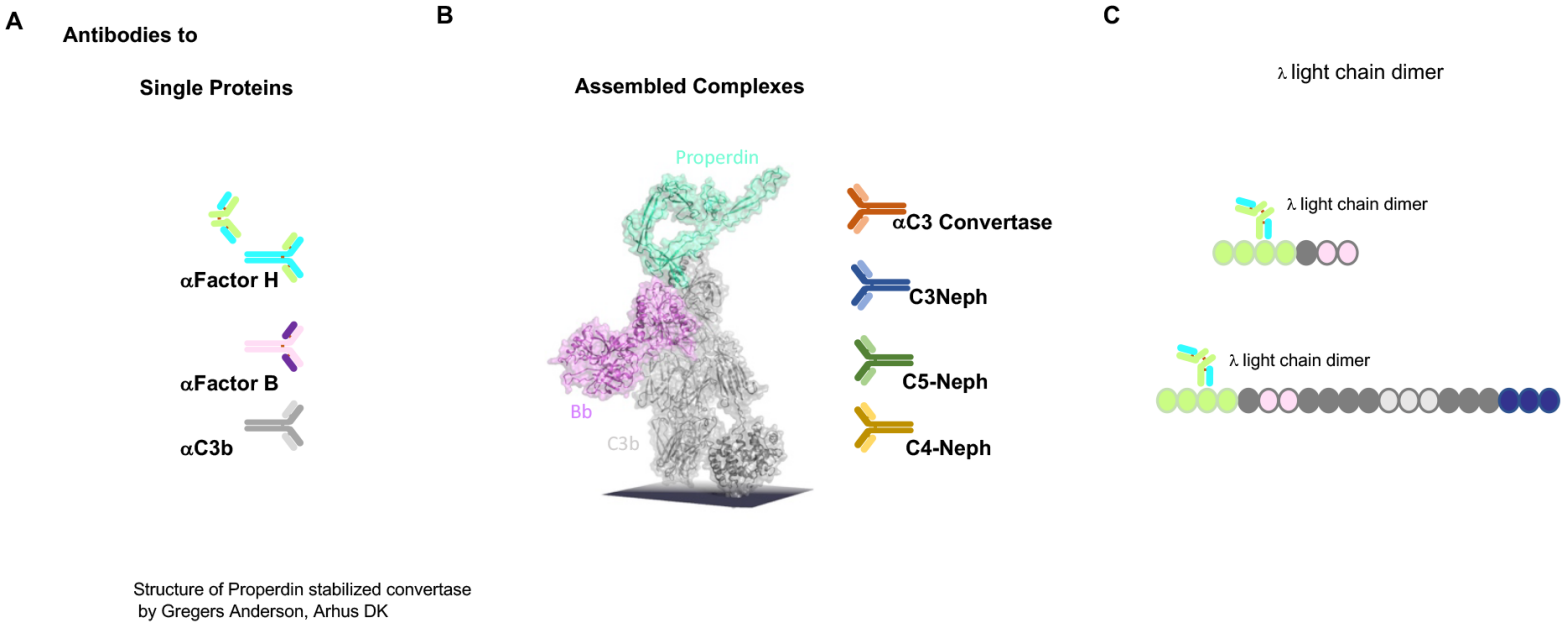
Autoimmune present targets of C3G. **(A)** Autoantibodies identified in C3G bind to different targets. Autoantibodies C3G bind to distinct complement proteins that either regulators of the AP C3 convertase or the elements of this enzyme. C3G-associated autoantibodies that bind single proteins, the regulators Factor H and FHL1, or the components that make up the convertase Factor B or C3. PLEASE add this after the elements of this enzyme. **(B)** The middle image shows the structure of the properdin-stabilized AP C3 convertase C3bBbP (structure by Gregers Anderson Aarhus, Denmark). These autoantibodies bind to neoepitopes exposed on the assembled convertase in the form of anti-C3 convertase antibodies and C3Nephritic Factor (C3NeF), as well as C5NeF. **(C)** Binding of nephritogenic λ light-chain dimers within the regulatory region of FHL1 and Factor H.

The autoimmune causes of C3G include antibodies directed against the individual complement proteins Factor H, C3b, and Factor B. In addition, autoantibodies such as C3 nephritic factors (C3NeF), which bind to neoepitopes of the central AP C3 convertase, are most common; C4NeF and C5NeF have also been described (45–48). Thus, the autoimmune scenarios include autoantibodies directed against individual complement proteins that either regulate or form AP C3 convertase and affect protein function or bind to and stabilize the AP C3 convertase complex (49) (**Figure 3**). Autoantibodies to Factor H have been described as IgG and IgM subclasses (50, 51). Both subclasses activate the classical pathway of complement.

The first example of an autoantibody in C3G, in the form of a Λ light-chain dimer targeting Factor H, has been well characterized with respect to AP C3 convertase amplification (see below) (52, 53).

The genetic alterations and autoimmune factors identified in C3G affect components that form the AP C3 convertase or regulators, including the emerging FHR modulators and the FHR5 activator. C3NeF factors bind to neoepitopes of the AP C3 convertase, stabilizing the enzyme and leading to increased activity and C3 consumption. C5NeF binds to the assembled AP C5 convertase. Although these causes are different and involve different genes, different proteins, and different protein targets, they deregulate AP C3 convertase and affect three steps of this pathway: fluid-phase activation via the tick-over mechanism, the action of surface-deposited C3 convertases, and the AP amplification loop. The overactive AP leads to C3 consumption, which explains the low plasma C3 levels. It also highlights the importance of AP action and shows that multiple regulators and modulators mediate precise AP convertase regulation.

### 3.4 Selected C3G cases with functionally characterized autoimmune or genetic causes provide insights into defective AP C3 convertase action in C3G

To uncover the common principle(s) of AP C3 convertase deregulation and to define how AP is deregulated in C3G, we selected five different cases: one autoimmune form and four familial cases with different genetic defects that presented the same renal pathology. For each scenario, the cause of the disease and the mode of action of the autoantibodies or the four different mutant proteins are well characterized (**Table 1**).

**Table 1:** Selected C3G scenarios, where the autoimmune or genetic causse and the underlying Selected C3G scenarios, where the autoimmune or genetic causse and the underlying pathophysiology are well evaluated. Complement parameters and clinical features of the C3G patients are presented.

Patients				Age at onset	EM	Plasma				Cause	Comment	Reference
**#1**	**Autoimmune**	**single patient**	**αFactor H Λlight chain**	56 y female	DDD	C3 low Factor B low	C3d up C5b-9up	**Λlight chain dimer**	–	defective Factor H & FHL1	respiratory infection	(54)
**#2**	**Genetic**	**Famiial**	**Factor HΔK224**	5 y female	DDD	C3 low Factor B low	C3d up C5b-9up	**g.DNA Δ57977-57969 p.ΔK224**	**hom**	defective Factor H & FHL1	C3Neph positive	(55)
**#3**				1 y female	DDD	C3 low Factor B low	C3d up C5b-9up	**g.DNA Δ57977-57969 p.ΔK224**	**hom**	defective Factor H & FHL1	C3Neph positive	(55)
**#4**	**Genetic**	**Familal**	**FHR1::FHR5 hybrid**	8 y female	DDD	C3 low Factor B low	C3d up C5b-9up	**chromsomal 146,751 bp deletion**a1: ΔFHR1-FHR4-FHR2	het	FHR1::FHR5 hybrid protein	FHR Multi-merization	(57)
**#5**				3 y female	DDD			a1: ΔFHR1-FHR4-FHR2a2: ΔFHR3-FHR1		FHR1::FHR5 hybrid protein		(57)
**#6**	**Genetic**	**Familal**	**FHR2::FHR5 hybrid**	18 y female	DDD	C3 absent Factor B low	C3d up C5b-9up	**chromsomal 24,804 bp deletion**	**hom**	FHR2::FHR5 hybrid protein	FHR Multi-merization	(58)
**#7**				15 y male		C3 absent Factor B low	C3d up C5b-9up	**chromsomal 24,804 bp deletion**	**hom**	FHR2::FHR5 hybrid protein	FHR Multi-merization	(58)
**#8**	**Genetic**	**Familal**	**C3923ΔDG**	28 y femal	DDD	C3 low Factor B low	C3d up C5b-9up	**C3 923ΔDG**	**het**	Dysregulated C3		(60)
**#9**				23 male		C3 low Factor B low	C3d up C5b-9up	**C3 923ΔDG**	**het**	Dysregulated C3		(60)
**#10**				16 male		C3 low Factor B low	C3d up C5b-9up	**C3 923ΔDG**	**het**	Dysregulated C3		(60)

The autoimmune case includes one patient with an autoantibody to Factor H and FHL1, and the four genetic cases include families with mutations in the Factor H gene, two with chromosomal deletions in the *FHR* gene cluster producing FHR2::FHR5 or FHR1::FHR5 hybrid proteins and one family with a C3 mutation (**Table 1**). Biopsies from all patients show C3G or, based on an early time of diagnosis, MPGNII/DDD. In addition, patient plasma showed an altered complement profile indicative of AP C3 convertase deregulation in the fluid phase C3b staining on glomerular surfaces. The induced local complement effector effects cause inflammation, cell proliferation, and deposition of activation products in the kidney, resulting in glomerular damage and C3G pathology.

For each scenario, the pathological cause is studied in detail and well-illustrated, and the mode of action of either the autoantibodies or the four mutant proteins is well characterized. These examples show on the one hand that an autoantibody and alterations of different complement genes share common pathophysiological principles and cause C3G.

The autoimmune case with autoantibodies to Factor H and FHL1 and each of the four genetic cases, one with mutations in the Factor H gene, two with chromosomal deletions in the FHR gene cluster producing FHR2::FHR5 or FHR1::FHR5 hybrid proteins, and the C3 mutation, define exactly how the autoimmune and genetic alterations cause deregulation of complement AP C3 convertase in the fluid phase and on glomerular surfaces. The deregulated AP C3 convertases cause C3 consumption, and strong local complement effects result in glomerular damage, inflammation, cell infiltration, proliferation, and activation, resulting in C3G pathology.

#### 3.4.1 Acquired Factor H–FHL1 defect (case #1)

A **λ** light-chain dimer that targets SCR3 in the regulatory regions of Factor H and FHL1 and blocks Factor H and FHL1 regulation was identified in a 52-year-old female patient with an upper respiratory tract infection prior to clinic presentation. The patient had elevated serum creatinine and urea levels. Renal biopsies showed an MPGNII/DDD pattern with intramembranous subendothelial deposits. Light microscopy showed increased matrix thickening and mesangial and endothelial proliferation, and electron microscopy showed dense deposits and the GBM appeared to be doubled. Immunohistochemical staining also showed C3 deposition, C5b-9/MAC staining, properdin deposition, and Factor H deposition detected along the GBM (53, 54).

Plasma complement analysis presented low levels of C3 and Factor B with normal levels of Factor H, Factor I, and C4. Hemolytic assays showed a severe reduction in AP-mediated activity with normal CP activity. The patients lacked classical C3NeF but presented a 46-kDa monoclonal **λ** light-chain dimer. This **λ**light-chain dimer binds to SCR3 in Factor H and FHL1, i.e., binds to the regulatory region and blocks the cofactor and degradation activity of both regulators. Thus, blockade of the regulatory action of Factor H and FHL1 results in potent AP convertase action and C3G.

#### 3.4.2 Genetic factor H–FHL1 defect (cases #2 and #3)

Two related patients were diagnosed with MPGNII/DDD at the ages of 5 and 1 year. Both patients had a homozygous deletion of three nucleotides in the Factor H gene (g.DNA Δ57967 -57969, c.DNA **Δ**743-745, p**.Δ**K224 in SCR4). At the time of diagnosis, both patients showed normal creatinine and albumin levels. Serum analysis revealed low C3, low Factor B, high C3d, and normal C4 levels. The patients were also positive for C3NeF. Factor H and FHL1 were present in the plasma, and functional analysis showed defective C3b binding, lack of cofactor, and degradation, accelerating activities but normal binding to endothelial cells (55, 56).

Thus, both the **λ** light-chain dimer targeting SCR3 and the homozygous **Δ**K224 mutation in SCR4 affect Factor H and FHL1 complement regulation. Patient #1, with the functionally inactivating Factor H/FHL-1 antibody, and patients #2 and #3, both expressing functionally defective Factor H and FHL-1, showed low C3 levels, AP dysregulation, and highly associated renal pathology in the form of C3G (at the time diagnosed as MPGN II/DDD).

#### 3.4.3 Complement overactivation by an FHR1::FHR5 hybrid protein (cases #4 and #5)

In an Indian family, the index patient was diagnosed with C3G at the age of 8 (57). Her 3-year-old sister presented with similar clinical symptoms. Complement analysis revealed low levels of C3 and Factor B and high levels of activation fragment Bb and sC5-9/MAC. Genetic analysis revealed a 146-kbp deletion on one allele spanning FHR1 exons v and vi via FHR2-FHR4- to the promoter region of FHR5. This constellation brought FHR1 exons 1–4 close to the FHR5 gene and resulted in the expression of an FHR1_1–3_::FHR5 hybrid protein. The second allele had the common FHR1–FHR3 deletion.

#### 3.4.4 Complement overactivation by an FHR2::FHR5 hybrid protein (cases #6 and #7)

In another familial case, the sister and brother of a European family developed the disease at the ages of 15 and 16 years, respectively, and their renal function declined over several years. At that time, both patients were diagnosed with MPGN II/DDD. Plasma diagnostics showed the absence of C3, low Factor B, high C3d, and C5b-9/MAC levels. APH 50 activity was reduced and C4 levels were normal. The underlying genetic defect was a homozygous deletion of a chromosomal region spanning the FHR2 gene exons iv and v to the promoter region of the FHR5 gene. This constellation results in the expression of an FHR2_1–2_::FHR5 hybrid protein (58). The hybrid proteins induced overactivation of the complement in the plasma, resulting in C3 consumption and low plasma C3 levels. Patient serum when mixed with NHS showed enhanced complement activity that consumed C3 within minutes, demonstrating a strong activating effect of the FHR2::FHR5 hybrid protein, which also formed large multimeric complexes in the plasma (58, 59).

Patients #4 and #5 and patients #6 and #7 from the latter two families expressing either FHR1_1–3_::FHR5 or FHR2_1–2_::FHR5 hybrid proteins developed C3G in the context of an intact *Factor H* gene and, thus, in the presence of Factor H and FHL1 in plasma, demonstrating that hybrid FHR proteins, which form large multimers in the plasma and contain the intact FHR5 or FHR1 backbone, enhance the amplification activity of FHR5, which enhances AP C3 convertase-mediated amplification.

#### 3.4.5 C3 mutation (cases #8–#10)

Another example of genetic C3G with a well-defined pathomechanism results from a heterogeneous mutation in the C3 gene, as shown for three members of a Spanish family (60). The patients showed normal C3 and C4 plasma levels, Factor B levels were at the low end, and hemolytic activity was reduced. All three patients had a six-nucleotide deletion on one allele of C3, resulting in a C3 protein missing two amino acids. C3923ΔDG could not be cleaved by the AP C3 convertase to C3b and represented the major circulating C3 protein in the plasma. Upon activation to C3b, C3b923ΔDG formed an active convertase that was resistant to decay by Factor H but was regulated by DAF. Activated C3923ΔDG and C3(H_2_O)923ΔDG were insensitive to Factor I-mediated cleavage in the presence of the cofactor Factor H. However, both mutant and wild-type C3 were cleaved and inactivated by MCP. This functional analysis of a mutant C3 protein highlights the role of AP convertase dysregulation in C3G pathology and suggests complex regulatory mechanisms of AP C3 convertase control.

These five different scenarios, representing one autoimmune case and four familial cases, show that defective AP C3 convertase control is triggered by different scenarios: i) by Factor H and FHL1 binding autoantibodies that block complement regulation (case #1), ii) by mutant Factor H and FHL1 proteins that lack complement regulation (cases #2 and #3), iii) by FHR1::FHR5 and FHR2::FHR5 hybrid proteins that form large multimers in the plasma and enhance FHR5 activation (cases #4, #5, #6, and #7), and iv) by a mutant C3 protein that is resistant to Factor H inactivation (cases #8, #9, and #10). These different pathological scenarios all trigger AP C3 convertase overactivity at the C3 tick-over step by deregulating surface-bound C3 convertase and inducing an overactive AP-mediated amplification loop. The four defects are manifested by low plasma C3 levels and lead to glomerular damage over time, resulting in C3G (or as diagnosed in previous years, MPGNII/DDD).

Factor H mutations have been reported in several C3G patients, and chromosomal deletions in the *CFHR* gene cluster have been described in members of six additional C3G families (56–60). These latter changes form FHR::FHR5, FHR::FHR1, or FHR5::FHR2 hybrid proteins. All hybrid proteins assemble into large multimeric protein complexes that enhance and overactivate AP C3 convertase, resulting in low plasma C3 levels. It is important to note that for the expression of FHR hybrid proteins, in C3G patients, altered complement plasma levels and renal pathology developed in the context of an intact Factor H gene, i.e., in the presence of the regulators Factor H and FHL1. Thus, hybrid FHR::FHR proteins deregulate AP C3 convertase and probably also C5 convertase, suggesting an important modulatory role of intact FHR proteins in proximal complement control.

## 4 Prevalence of C3G and data on C3G frequency in five Indian hospitals

The prevalence of C3G is estimated to be one to two cases who attend the clinic per one million population (4). More detailed regional studies showed point prevalence in different European countries and in the USA. In a French cohort, the assessed cohort prevalence is 0.14 per 10,000 (30), and in the UK and Irish cohorts, there are 1.3 and 1.1 cases per 10,000 respectively. Moreover, frequencies of up to five patients per million have also been reported. Data from the USA range from 1 to 2–3 cases and even up to 5 cases per 1,000,000 population (5).

Such differences can be explained by differences in diagnostic routines and workups. A worldwide comparison may include additional parameters such as ethnic variability, differences in disease awareness, access to healthcare, availability of full complement diagnostics, genetic testing, and availability of detailed biopsy evaluation that may further influence the prevalent numbers.

For example, the *FHR* gene cluster, which underlies a region of chromosomal instability and a hotspot for structural rearrangements, is associated with several disorders, including C3G and aHUS, as well as AMD. Homozygous FHR1/FHR3 deficiency is a multifaceted condition. This deficiency is associated with kidney diseases such as C3G, IgAN, DEAP-HUS, and SLE; has a protective effect (IgAN and AMD); and causes risk scenarios (DEAP-HUS, SLE). Addiitionally, homozygous FHR1–FHR3 deficiency is present in healthy individuals and shows strong ethnic variations: in about 6% in Caucasian, approximately 17% in Asian, and approximately 27% in African populations (61–64).

### 4.1 C3G prevalence in five Indian nephrology centers

There are insufficient data on the prevalence (number of cases per 100,000 people) of C3G in India. However, the Indian C3G family with an FHR1::FHR5 hybrid protein, the high frequency of FHR1–FHR3 deletions in the Indian population, and the high prevalence of autoimmune DEAP-HUS in India suggest a higher frequency of FHR gene cluster variations that may cause C3G. To prove this hypothesis, a retrospective analysis was conducted in five nephrology centers from different regions of India. The prevalence of C3G was determined from past available clinical records. The retrospective analysis includes C3G patients whose diagnosis was made based on renal biopsy and was further supported by clinical parameters and, when available, laboratory analyses (**Table 2**).

**Table 2:** Prevalence of C3G in nephrology units from five hospitals in India.

		Hospital		Number of admitted patients with acute and chronic kidney disease in 2 years		Number of C3G patientsdiagnosed in two years	Age group (years)		Gender		Prevalence of C3G (per 10,000 AKI patients)	
**1**		**Max Hospital, Delhi**		**2,500**		**2**	35, 68		2 males		16	
**2**		**JIPMER, Pondicherry**		**8,250**		**2**	24, 35		1 male 1 female		4.85	
**3**		**DY Patil Hospital, Pune**		**NA**		9	16, 24, 25, 28, 29, 30, 35, 45, 61		8 males 1 female		–	
**4**		**KEM Hospital, Pune**		**NA**		5	13, 16, 18, 28, 31, 33		4 males 2 females		–	
**5**		**Jaypee Hospital, Noida**		**4,000**		**3**	10, 16, 21		2 males 1 female		15	
		**Total (from 3 centers)**		14,750*		7*	Mean 29.13				12.15*	

*Data from centers 1, 2, and 5 are included.Most patients diagnosed with C3G were adults, with an average age of onset ranging from 20s to 60s, with a mean value of 29.13 years. The age groups varied among the five hospitals. There was a significant male predominance with 89 % of the patients being. However, further analysis with more patients and more hospitals is needed. NA signifies information is not available.

This information provides insight into the prevalence of this serious kidney disease in India and may further provide a basis for a detailed analysis of the underlying genetic and autoimmune causes.

Patients diagnosed with C3G over a period of 2 years were included. This retrospective evaluation showed that between two and nine cases of C3G were diagnosed in the five nephrology units over a 2-year period (**Table 2**).

For each of the five centers, the diagnosis of C3G is based on kidney biopsies using immunohistology combined with electron microscopy.

Despite the mentioned limitations, the data from the five nephrology units suggest that the prevalence of the disease in India is at least comparable to that in other countries and maybe higher.

This may propose that genetic, autoimmune, and environmental factors influence the incidence and prevalence of the disease in India. Additional access to healthcare or disease awareness may also influence the presentation of patients. Comparing these frequencies between different regions of India will also be informative and provide more accurate data on disease frequencies between states and ethnic groups. It is likely that more C3 patients will be identified in more states with increased and more accurate diagnosis.

## 5 Treatment of C3G

There is currently no effective therapy for C3G, and although it is an AP complement disease, no complement inhibitor is approved for C3G therapy. The off-label use of eculizumab has been performed with mixed results. The therapy was effective in some but not all patients (65).

C3G patients often receive supportive care, which often includes blood pressure control with ACE inhibitors or angiotensin II receptor blockers (ARBs) to reduce proteinuria and slow down kidney damage. Corticosteroids and immunosuppressive drugs such as mycophenolate mofetil, cyclophosphamide, and rituximab are used in some situations. Corticosteroids may be used to treat inflammation and proteinuria although their effectiveness may vary (65–70).

Immunosuppressive therapy is used to reduce the production of autoantibodies and to reduce the inflammatory response.

Some patients are also treated with plasma exchange or plasmapheresis, which is used to replace defective complement proteins or to remove autoantibodies and other forms of debris. Long-term experience with plasma exchange was gained, for example, with patient #1 and patients #2 and #3 from family 1 (71). This therapy was tolerated for several years, and later on, the patients developed autoantibodies and one was switched to eculizumab therapy (72, 73).

### 5.1 Mechanistic insights targeting complement inhibitors

**Figure 4:**
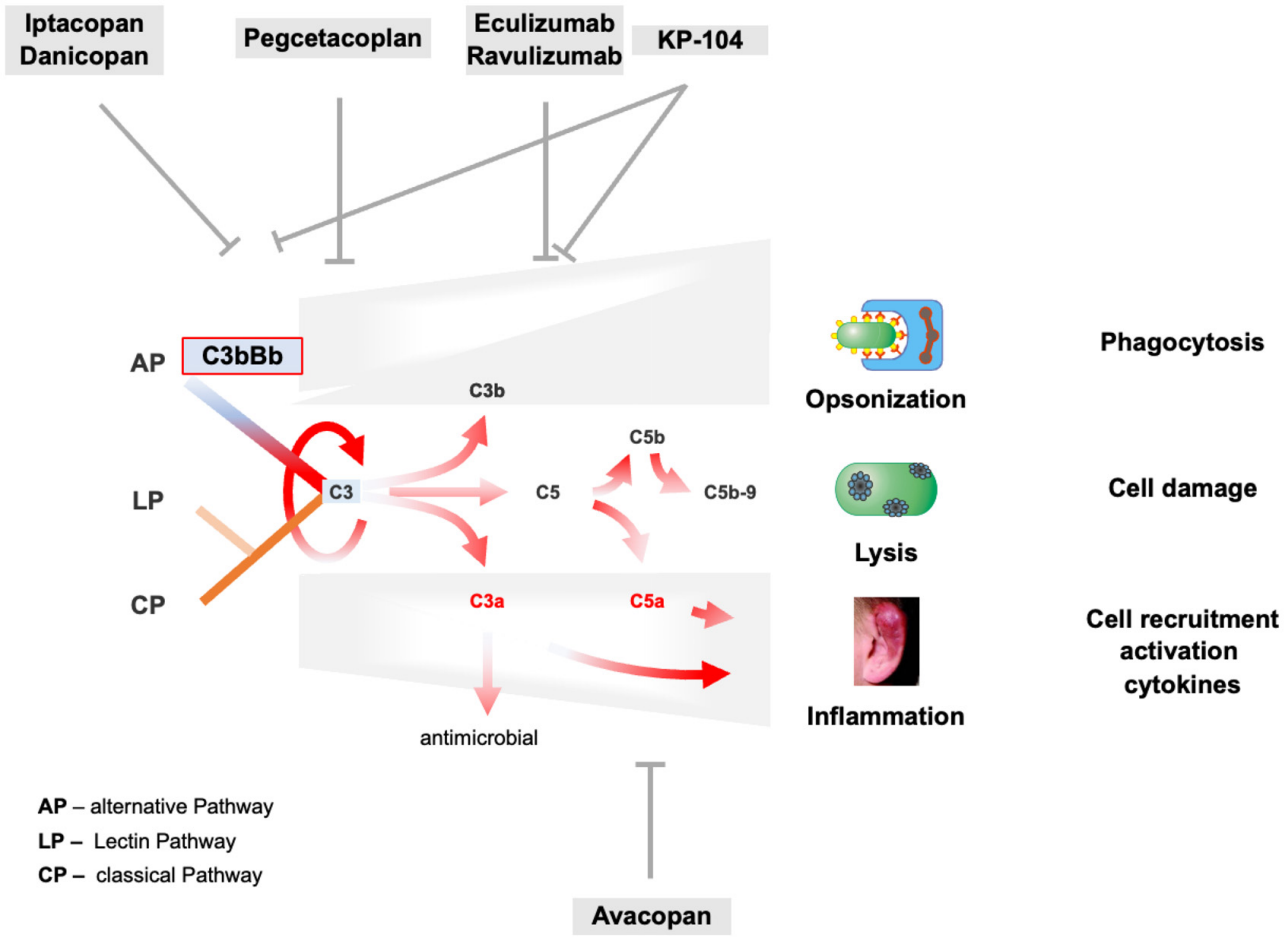
AP C3 convertase and AP amplification loop dysregulation trigger C3G pathogenesis and target profiles of complement inhibitors. The tick-over, the AP C3 convertase, and the AP-mediated amplification loop are deregulated in C3G as presented for selected and well-characterized cases in Chapter 3.4. AP overactivation induces strong complement activation shown by the red arrows and induces all effector levels of the complement. Consequently, C3a- and C5a-mediated cell recruitment and inflammation, C3b-assisted opsonization, and C5b-9-mediated lysis are turned on and are overactive. On the top and bottom, major complement inhibitors are evaluated in phase II or III clinical trials, including their corresponding targets.

The examples reported for the 10 well-studied C3G patients, summarized in **Table 1**, show common mechanisms in C3G patients with autoimmune and different genetic defects. Defective AP regulation occurs during the C3H_2_O tick-over, the AP C3 convertase, and the AP-driven amplification loop. Defective complement regulation during the early, proximal stage switches on all four effector pathways (**Figure 4**), resulting in the release of anaphylatoxins C3a and C5a, causing cell recruitment and inflammation, C3b-enhanced opsonization, AP C3 convertase formation, AP amplification loop, and TCC/C5b-9-facilitated cell lysis. With all effector loops turned on, it is challenging to predict the correct target and the most promising complement inhibitor for intervention and complement therapy. Consistent with this concept, the use of the terminal complement inhibitor eculizumab in C3G showed that inhibition of the terminal pathway was effective in some but not all C3G patients.

Currently, several complement inhibitors are being evaluated in phase II and III clinical trials, targeting different complement proteins and affecting different levels of the complement cascade (**Figure 4** and **Table 3**).

**Table 3:** Complement inhibitors in clinical trials for C3G (as of 3 June 2024).

	Compound	Target	Drug type	Application	Clinical phase	Study number
1	Iptacopan	Factor B	Small compound	p.o.	2, 3	NCT03955445 NCT04817618
2	Danicopan	Factor D	Small compound	p.o.	2	NCT03368236 NCT03124368
3	Pegcetacoplan	C3	Peptide	s.c.	2, 3	NCT05809531 NCT05067127
4	KP104	C3 convertase and C5	Fusion protein mAB – Factor H**_1–5_**	i.v., s.c.	2	NCT05517980
5	Eculizumab	C5	mAB	i.v.	1	NCT01221181
6	Avacopan	C5aR1	Small compound	p.o.	2	NCT03301467

### 5.2 Targeted complement therapeutics in development

The detailed understanding of the pathomechanism of C3G and the knowledge of which complement pathways are involved initiated clinical trials with targeted therapy. In recent years, several complement inhibitors have been developed and are being tested in clinical trials against C3G. Additional inhibitors are under development and in the early stages of evaluation. Inhibitors that are evaluated in advanced phase II and III trials for C3G are presented in **Table 3**.

Inhibitors target the complement at different levels or steps; some block the complement in the proximal, others in the distal phase, and still others target specific pathways such as the alternative pathway or the inflammatory C5a axis. Several complement inhibitors are approved, but not yet for C3G, and several inhibitors are in clinical trials for C3G (7, 8, 74–76).

Types of inhibitors: Complement inhibitors include several types of substances, such as monoclonal antibodies, nanobodies, small chemical compounds, inhibitory RNAs, pathogen-derived proteins, and recombinant endogenous regulators. In addition, biosimilar products have already been approved for off-patent inhibitors. Specific targeting of complement will allow for high selectivity and improved safety.

#### 5.2.1 Iptacopan

Iptacopan is an orally administered selective inhibitor of the protease Factor B. Iptacopan binds specifically to Factor B and blocks proteolytic cleavage. Iptacopan blocks C3 tick-over activation, AP C3 convertase action, and the amplification loop (77, 78). CP and LP activation remain intact. Iptacopan has been evaluated in phase I and phase II studies demonstrating safety, tolerability, and renal function stabilization. Phase III trials for C3G are ongoing (**Figure 4**).

#### 5.2.2 Danicopan

Danicopan is an orally administered small molecule inhibitor that binds with high affinity to protease Factor D and blocks proteolytic activity. Danicopan blocks the cleavage of Factor B and, thus, the formation of the active AP C3 convertase (79, 80). Two phase II studies have demonstrated the safety and tolerability of danicopan. Danicopan recently received approval in Japan for combination therapy with eculizumab.

#### 5.2.3 Pegcetacoplan

Pegcetacoplan, also a chemical inhibitor, is administered by s.c. injection. This small molecule binds selectively to C3 with high affinity and blocks the C3 convertases of both AP and CP/LP (80–82). Pegcetacoplan is approved for the treatment of PNH and geographic atrophy. Phase II trials of pegcetacoplan in C3G are ongoing, and results have recently been published. Phase III trials are ongoing.

#### 5.2.4 KP-104

KP-104 is a dual-acting inhibitor combining a C5 targeting mAB with the regulatory fragment of the complement inhibitor Factor H (83, 84). With this profile, KP-104 targets the distal complement by C5 blockade and further controls AP action via the tick-over, AP C3 convertase, and AP-mediated amplification loop.

#### 5.2.5 Eculizumab

Eculizumab was the first approved complement inhibitor and is currently used in saeveral diseases (85–88). This monoclonal antibody targets complement C5 and inhibits the formation of anaphylatoxin C5a and the membrane attack complex. Eculizumab has been used off-label in C3G patients (Ref). Eculizumab was effective in some but not all C3G patients. This suggests that terminal complement inhibition by blocking C5a and lytic effector functions induced by all three activation pathways is not sufficient for C3G therapy.

The use of eculizumab in C3G patients demonstrated that terminal pathway inhibition is effective in some but not all C3G patients.

#### 5.2.6 Avacopan

Avacopan is also a small chemical compound that binds to C5R1/CD88 and specifically blocks C5a-mediated inflammation (88). Avacopan leaves all three activation pathways intact and does not interfere with AP tick-over, AP C3b-mediated effects, or AP-mediated amplification. Avacopan is approved for the treatment of ANCA vasculitis and phase 2 clinical trials in C3G are ongoing (81).

#### 5.2.7 Rationale for C3G therapy with recombinant Factor H (CPV-104)

An interesting and promising option for C3G therapy is the use of endogenous regulators or inhibitors to restore imbalanced deregulated complement. Defective Factor H complement regulation, either by autoantibodies (patient #1) or by gene mutations (patients #2 and #3), results in C3G. For these patients, the substitution of defective Factor H may be an attractive option to rebalance deregulated complement in the patient’s plasma, especially considering the potent regulatory effects of Factor H within the complement and the additional functions of its regulator in inflammation.

Full-length Factor H can be expressed with high yields in a moss-based system. Purified moss-expressed Factor H, i.e., CPV-104 after glycosylation, retains all regulatory functions and shows long-term plasma bioavailability *in vivo*. The therapeutic potential of glycosylation-optimized CPV-104 is being pursued by eleva GmbH and clinical trials are pending (89).

## 6 Discussion

C3G is caused by dysregulation of proximal complement. The examples summarized for the autoimmune case and the four genetic familial cases together with other cases evaluated in detail show that C3G complement dysregulation mainly affects the initial steps of the AP, the fluid-phase C3 tick-over activation, the AP-mediated C3 convertase surface action, and the AP amplification loop. This deregulation results in C3 consumption in the circulation, low plasma C3 levels, low Factor B levels, and glomerular damage. The LP and CP pathways are not affected and remain intact.

Based on the heterogeneity of C3G, many, but not all, C3G patients have reduced or low or absent serum C3 levels and normal C4 levels. Similarly, the activation markers C5b-9/MAC and Bb, Ba, and C5a levels are elevated in many, but not all patients. Thus, C3G is a heterogeneous disease as evidenced by the variable changes seen in renal biopsies. Additional genetic and autoimmune causes are likely.

### 6.1 Factor H vs. FHL1

The scenarios presented in Sections 3.4.1 and 3.4.2 include autoimmune and genetic scenarios of C3G in which the regulatory actions of both Factor H and FHL1 are blocked. Apparently, Factor H regulation is more relevant than FHL1 control. The 13-month-old Native American of the Sioux tribe, who was diagnosed with membranoproliferative glomerulonephritis, lacked Factor H in the plasma. Genetic analysis revealed frameshift Cys mutations in SCR9 and SCR16, leading to a block in protein secretion and intracellular accumulation of Factor H. Consistent with the C-terminal position of the mutated Cys residues, this patient expressed FHL1 in the circulation (31, 32). This highlights that in the glomerular environment, the complement control of Factor H is more relevant than that of FHL1.

### 6.2 C3G vs. aHUS

Both the kidney diseases C3G and aHUS are prominent examples of complement disorders, and the pathophysiology due to defective complement is well understood. Interestingly, the same genes Factor H, C3, Factor B, and Factor I are involved in both diseases, but the mutations cause different pathologies. For example, different functional regions of Factor H are affected by genetic mutations and autoantibodies. The N-terminal regulatory region of Factor H is affected in C3G, and the C-terminal discriminatory region is affected in aHUS. This demonstrates that alterations in different functional segments of this regulator are associated with different diseases and highlights the importance of fine-tuned complement regulation. In C3G, chronic AP dysregulation is often correlated with low C3 plasma levels, and in aHUS, acute scenarios result in endothelial damage. Deregulation of the proximal complement of the early AP C3 convertase leads often to C3 consumption in the circulation, explaining the low plasma C3 levels and the increase in complement activation products such as C3d, Ba, Bb, and C5b-9/MAC. This results in local glomerular damage. In C3G, complement dysregulation occurs at the initial, proximal level of the AP, and consequently, all four effector levels are active. In aHUS, dysregulation occurs at the late step in the form of C5b-9/MAC-mediated endothelial damage in small glomerular vessels. This explains the efficacy of the terminal complement pathway inhibitors eculizumab and ravulizumab.

Members of the Indian C3G family presented in Chapter 3.4 show compound heterozygous changes in the FHR gene cluster, a chromosomal deletion resulting in an FHR1::FHR5 hybrid gene on one allele and an FHR1::FHR3 deletion on the second allele. This scenario, and since chromosomal FHR1::FHR3 deletions are common in India, and furthermore, the high frequency of the FHR1–FHR3-associated autoimmune form of DEAP-HUS in the country, suggests that chromosomal FHR gene cluster rearrangements may be common in the Indian population. By analyzing the prevalence of C3G in five nephrology centers, we aimed to provide the first indication of the frequency of C3G in India. These data are consistent with other cohort studies. The data clearly show limited number of five nephrology centers does not yet allow precise statistical data on the prevalence of C3G in India. The data clearly show that C3G is present in the country. It will be interesting to extend such studies to more nephrology centers getting data from more patients and different locations in the country and receiving more precise information on the *FHR* gene status of C3G patients in India.

At present, there is no approved complement therapy for C3G. Complement dysfunction leading to glomerular pathology is now well understood. In most C3G patients early, proximal complement is deregulated at the C3 level, and all effector pathways are activated. This may explain why C5 inhibition by eculizumab was not effective in all C3G patients and shows the rationale for focusing on the early steps of AP activation, targeting the proximal AP to rebalance the cascade. Several complement inhibitors are being evaluated in clinical trials, including phase II and phase III trials.

## 7 Outlook

The intensive research in the field of C3G, along with the evaluation of several complement-targeting therapeutics and inhibitors for C3G that are currently in phase II and phase III trials, gives a very promising situation, for providing effective treatment for this kidney disease in the near future and giving hope to patients and their families.

However, even though inhibitors have been approved for C3G, some questions remain: Will complement inhibition alone be effective? Will complement inhibitors be used in combination with immunosuppressive or glucocorticoid therapy or in combination with MMF or is there a need to combine two or even more complement inhibitors to block all effector pathways? Duration of therapy is also relevant, will treatment be lifelong or can an inhibitor be tapered?

Clearly, complement diagnostics need to be improved. Guidelines such as KDIGO recommend, in addition to biopsies, detailed complement profiling of the plasma and urine of C3G patients, including APH50 activity, complement protein levels, activation products, and autoantibodies in combination with genetic testing. With several complement therapeutics expected to be approved soon, improved complement profiling will help clinicians make personalized therapy decisions.

Other questions that will arise from long-term studies include whether proximal complement inhibition over many years will be as well tolerated as distal blockade, what the adverse effects will be, including infections with pathogenic microbes, and whether these inhibitors will reveal new and previously unknown regulatory loops in the complement cascade. Also, whether the same vaccination will be required for each complement inhibitor, which pathogenic microbes’ vaccination is most relevant, or whether vaccination is not required for regulators that restore endogenous complement.

In summary, complement inhibitors will most likely be approved for C3G in the coming years, improving the quality of life for C3G patients and their families.
